# Application of Traditional Chinese Medicines in Postoperative Abdominal Adhesion

**DOI:** 10.1155/2020/8073467

**Published:** 2020-04-26

**Authors:** Fuling Wu, Wenqin Liu, Haixing Feng, Li Long, Lianbing Hou, Chuqi Hou

**Affiliations:** ^1^Department of Pharmacy, Nanfang Hospital, Southern Medical University, Guangzhou, Guangdong, China; ^2^School of Pharmaceutical Sciences, Southern Medical University, Guangzhou, Guangdong, China

## Abstract

Adhesion is a frequent complication after abdominal surgery. Although various methods have been applied to prevent and treat postoperative abdominal adhesion (PAA), few modern drugs designed for clinical applications have reached the expected preventive or therapeutic effect so far. There is an imperative to develop some new strategies for the treatment of PAA. Traditional Chinese medicine (TCM) has been widely practiced for thousands of years and played an indispensable role in the prevention and treatment of diseases. Modern medicine researchers have accepted the therapeutic effects of many active components derived from Chinese medicinal herbs. The review stresses the most commonly used TCM treatment, including Chinese medicinal herbals and monomers, TCM formulas, and acupuncture treatment.

## 1. Introduction

Postoperative abdominal adhesion (PAA) is one of the most common complications, which account for more than 90% of patients who had abdominal surgery, and approximately 30% of patients require the second surgery for PAA [1, 2]. Complications generated by PAA can result in numerous problems, such as chronic abdominal pain, small bowel obstruction, infertility in women, and even the secondary surgery [3, 4]. Furthermore, 40% of intestinal obstruction is caused by PAA [5].

PAA refers to the pathological connection between the omentum, abdominal organs, and abdominal wall after surgery. The formation of PAA is a complex process that involves immune activation, inflammatory response, fibrinolysis imbalance, oxidative stress, collagen deposition, peritoneal tissue repair, and other biochemical events [2, 6]. Abdominal surgery injury stimulates immune activation and inflammation response immediately. The interaction of various inflammatory cytokines causes the overproduction and accumulation of reactive oxygen species. Oxidative stress stimulates the formation of PAA. On the other hand, fibrin deposition contributes to the injury healing to a certain degree. However, disorders of the fibrinolytic process, such as the imbalance of tissue-type plasminogen activator (t-PA) and plasminogen activator inhibitor-1 (PAI-1), could result in the formation of abdominal adhesion [7]. Collagen deposition is similar to that of fibrin. The imbalance of the proenzymes of matrix metalloproteases (MMPs) and tissue inhibitors of MMPs (TIMPs) could also cause the formation of abdominal adhesion [8].

Various methods had been reported to prevent adhesion formation after surgery. Some intraoperative techniques, such as avoiding unnecessary peritoneal dissection and serosal tissue drying, reducing use of foreign bodies, and using starch-free gloves and laparoscopic procedure, are the basic methods for the prevention of PAA [9]. Mechanical barriers prevent postoperative adhesion formation by keeping peritoneal surfaces separate during the injury healing, such as hyaluronic acid (Sepracoat®), icodextrin (Adept®), sodium hyaluronate-carboxymethylcellulose (Seprafilm®), and oxidized regenerated cellulose (Interceed®) [4, 10, 11]. However, application locations of barriers need to be judged subjectively and accurately by the surgeons, which is difficult for some surgeons [12]. On the other hand, some synthetic materials failed because they initiate an inflammatory response or even cause abnormal adhesion around the edges of the material [13]. Chemical agents are generally studied to prevent the formation of abdominal adhesion, such as anti-inflammatory drugs, antioxidants, fibrinolytic agents, and selective immunosuppressors [14]. However, there are several side effects still to be considered, such as gastrointestinal bleeds caused by nonsteroidal anti-inflammatory drugs and hemorrhagic complications caused by fibrinolytic agents [8]. Although lots of great achievements have been gained in the battle against PAA during the past decades, PAA is still a question needed further research.

Traditional Chinese Medicine (TCM) has more than 2,000 years of history and has been confirmed in a range of clinical practices [15–17]. According to the basic theory of TCM, qi, blood (xue), yin, and yang are four essential physiological components in the human body. Qi and blood are two substances that make up the human body and maintain its life activities. Human health constitution could contribute to the balance between the qi and blood conditions [18]. The common symptoms of PAA are as follows: distending pain or a tingling sensation in a fixed position, dim complexion, depression, indigestion, abdominal bloating, constipation, and intestinal obstruction. It is categorized as “accumulation,” “Guge,” and “intestinal knot” in the TCM. According to the clinical analysis, the pathological bases of PAA are attributed to Qi stagnation, damp stagnation, and blood stasis [19]. Under the guidance of TCM pharmaceutical theory, therefore, herbs that activate blood and dissolve stasis, invigorate Qi, and strengthen the spleen are chosen to treat patients with PAA. In this review, we looked back on the applications of TCM in the treatment of PAA.

## 2. Chinese Medicinal Herbs and Monomers

### 2.1. *Salvia miltiorrhiza* Bunge

*Salvia miltiorrhiza* Bunge, a well-known traditional Chinese medicinal plant with thousands of years of clinical application, is a classical herb that promotes blood circulation and removes blood stasis [20]. It has several functions, such as prevention and treatment of heart diseases, treatment of asthmatic, oncotherapy, and others [20–22]. Salvianolate is a primary active component of *Salvia miltiorrhiza* Bunge. Sui et al. [23] have reported that salvianolate obviously decreases the levels of interleukin-1beta (IL-1*β*) and tumor necrosis factor-alpha (TNF-*α*) and inhibits the formation of adhesion tissue after operation. The study indicated that treatment with 24 mg/kg or 48 mg/kg salvianolate for 3 days can significantly reduce the extent of PAA in the experimental adhesion model of rats. Tanshinone IIA is another major extract obtained from the root of *Salvia miltiorrhiza* Bunge [24]. It has been found that treatment with 10 mg/kg or 2.5 mg/kg tanshinone IIA for 7 days could increase fibrinolysis activity in the peritoneal fluid and the rate of t-PA and PAI-1 and, at the same time, decreases the expression of cyclooxygenase-2 (COX-2) in the experimental adhesion model of rats [25]. Therefore, it is another potential compound in *Salvia miltiorrhiza* Bunge for the treatment of PAA.

### 2.2. Resveratrol

Resveratrol, one of the most important naturally active polyphenols, has some proven effects, such as antioxidant, anti-inflammatory, and antitumour [26, 27]. Resveratrol not only reduces oxidative stress and inflammatory response but also modulates immune responses [28, 29]. These effects make it possible for the treatment of PAA. Wei et al. [30] found that treatment with 20 mg/kg or 40 mg/kg resveratrol for 8 days can prevent PAA in a rat model, which may be related to the inhibition of the inflammatory response in the injured peritoneum. Resveratrol may be an effective compound treated with PAA, which is worth further study.

### 2.3. Honokiol

Honokiol, a biologically active substance derived from the bark of magnolia trees, has been shown to exert significant anti-inflammatory, antioxidative, antitumour and neuroprotective effects [31–33]. Agacayak et al. [34] found that treatment with 1 mg/kg honokiol for 14 days was effective in prevention of PAA in a rat model. It may be related to its effects of anti-inflammatory and antifibrosis because the inflammation score and fibrosis score are significantly lower in the honokiol group compared with the saline group in the study. However, more evidence should be found to support the use and quantity of honokiol and the exact role of honokiol in the treatment of PAA.

### 2.4. Berberine Hydrochloride

Berberine hydrochloride is a natural plant alkaloid isolated from various Chinese medicinal herbs, such as Huanglian (*Coptidis Rhizoma*) and Huangbo (*Phellodendri Chinrnsis Cortex*). It has some effects, such as antitumour, anti-inflammatory, and immunoregulatory [35]. Recently, it has also been reported that berberine hydrochloride could be a promising strategy to prevent PAA as it has anti-inflammation effect [36]. The study has shown that treatment with 1.5 mg/ml or 0.75 mg/ml berberine hydrochloride for 14 days can significantly prevent the formation of abdominal adhesion in rats, which may downregulate intercellular adhesion molecule-1 (ICAM-1) and inhibit the transforming growth factor-activated kinase 1 (TAK1)/c-Jun N-terminal kinase (JNK) and TAK1/nuclear factor κB (NF-κB) signaling after abdominal surgery. It has been proven that berberine hydrochloride can prevent adhesion formation after surgery and has potential application value.

### 2.5. Rhynchophylline

Rhynchophylline is a significant pharmacological active component isolated from Gouteng (*Uncariae Ramulus cum Uncis*) [37]. Song et al. [38] have reported that treatment with 6 mg/kg or 12 mg/kg rhynchophylline for 10 days can relieve the PAA in rats with evidence indicating that rhynchophylline downregulates inflammatory cytokines and the expression of connective tissue growth factor (CTGF). The potential mechanism of rhynchophylline in the treatment of PAA may relate to the inhibition of the transforming growth factor-*β*1 (TGF*-β*1)/Smad signaling pathway. It brought out a novel therapeutic approach for the development of clinical application for PAA.

### 2.6. Breviscapine

Breviscapine is a main component of purified flavonoid extract from Dengzhanxixin (*Erigeron breviscapus*) [39]. Previous studies have shown that breviscapine exhibited the ability of antioxidant, anti-inflammatory, and antifibrosis on many diseases [40, 41]. Zhang et al. [42] indicated that treatment with 20 mg/kg or 40 mg/kg breviscapine for 10 days is effective in the prevention of the formation of PAA in rats. The study indicated that it not only inhibits inflammation but also upregulates peritoneal fibrinolysis activity and regulates the TGF and/or Smad signaling pathway. However, more clinical research data are needed to support its application for the prevention of PAA.

### 2.7. *Bletilla striata*

*Bletilla striata*, as a traditional Chinese herb, has been applied to treat alimentary canal mucosal damage, ulcers, bruises, and burns for thousands of years [43, 44]. Previous studies have exhibited the antioxidant, anti-inflammatory, antitumour, antiviral, and antibacterial capabilities of *Bletilla striata* [45, 46]. Liu et al. [47] have reported that treatment with 15% *Bletilla striata* extraction solution for 7 days can prevent PAA in an abrasion-induced model of rats, owing to reducing the expression of the important substance which increased in PPA, such as IL-6, IL-17F, TNF-*α*, TGF-*β*1, and collagen. It indicates that *Bletilla striata* has obvious preventive effect on PPA through inhibiting inflammatory response after surgery.

### 2.8. Ligustrazine

Ligustrazine is a major active alkaloid monomer, which is extracted from the dried root of Chuanxiong (*Ligusticum chuanxiong* Hort.) [48]. It has a wide range of pharmacological activities, with high safety and few side effects, such as antioxidant, anti-inflammatory, antifibrosis, and antiapoptosis [49]. Yan et al. [50] and Zhang et al. [51] have reported that ligustrazine is a potent drug prevented PAA. The studies found that treatment with 30 mg/kg or 60 mg/kg ligustrazine for 10 days could regulate the TGF/Smad signaling pathway in the experimental adhesion model of rats; moreover, it could reverse the induction of fifibrosis-related cytokines in rat peritoneal mesothelial cells (RPMCs). Furthermore, ligustrazine nanoparticles nano spray has been researched and developed, which could be applied as a novel intervention for PAA [52].

### 2.9. Curcumin

Curcumin is an active principal curcuminoid of turmeric (*Curcumae Longae Rhizoma*) [53]. Curcumin is known recently to have antioxidant, anti‐inflammatory, antimicrobial, and immune-regulatory effects, which make it play an important role in prevention and treatment of various illnesses, such as cancer, autoimmune, cardiovascular diseases, and diabetic [54, 55]. Futhermore, curcumin has also been shown to increase fibrinolytic activity and cell migration towards the wound area, which makes it possible to be safe and effective in the prevention of PAA [56]. It has shown that treatment with 10 mg/kg curcumin for 15 days can significantly prevent PAA [57]. However, further studies are needed to show the most suitable and effective dose of curcumin for PAA in clinics.

### 2.10. Silymarin

Silymarin is a complex mixture of polyphenolic molecules derived from the seeds of the milk thistle plant Shuifeiji (*Silybum marianum*), which contains one flavonoid and a family of flavolignans including silychristin, silydianin, isosilychristin, silybin A, silybin B, isosilybin A, and isosilybin B [58]. Silymarin has anti-inflammatory, antifibrotic, and antioxidant properties [58, 59]. Previous studies have shown that 3 ml, 1% or 5% concentration, of the silymarin extract was administered into the abdominal cavity for 14 days, which is effective to prevent PAA [60, 61]. It may be attributed to the inhibition of collagen overproduction and accumulation, and complete lysis of fibrin in the experimental adhesion model of rats. Although it has been used as a medical herb from as early as the fourth century B.C., the effective administration and the dosage are difficult to be reproduced in humans due to its low bioavailability [62]. The problems need more data from clinical trials to solve.

### 2.11. Gallic Acid

Gallic acid, a trihydroxybenzoic acid, is widely found in tea leaves and some plants, such as grape seed, rose flowers, sumac, oak, and witch hazel [63, 64]. Gallic acid is a viable wound healing agent and a potential intervention to treat wounds through accelerated cell migration [65]. Gallic acid interferes with various inflammatory pathways and reduces the expression and activity of proinflammatory cytokines. Gallic acid inhibits the expression of nuclear transcription factors, such as NF-*κ*B and signal transducer and activator of transcription 3 (STAT3), and downregulates the expression of matrix metalloproteinase 2 (MMP2) and matrix metalloproteinase 9 (MMP9) [66, 67]. Therefore, gallic acid may be a promising drug for preventing intra-abdominal adhesions. Wei et al. [67] have reported that treatment with 100 mg/kg or 150 mg/kg gallic acid for 7 days attenuates PAA by inhibiting inflammatory response in the experimental adhesion model of rats.

### 2.12. Green Tea

As we all know, green tea or green tea infusion has health benefits owing to the presence of antioxidant phenolic compounds [68, 69]. Green tea has some effects, such as antiphotoaging, stress resistance, and neuroprotective and autophagy [70]. Parsaei et al. [71] have reported that treatment with 4% green tea extract for 14 days reduces the event of PAA in an animal model for it has antioxidant and anti-inflammatory effects which prevent production and accumulation of collagen. However, there are some problems need to be solved, for example, whether green tea could reach the expected preventative effect in the clinical application.

## 3. TCM Formulas

### 3.1. The Intestine Function Recovery Decoction

The intestine function recovery decoction (IFRD), a famous herbal formula consists of nine different herbs: Dangshen (*Codonopsis pilosula Franch.*), Baizhu (*Atractylodes macrocephala Koidz.*), Taoren (*Prunus persica Batsch*), Chishao (*Paeonia lactiflora Pall.*), Zhiqiao (*Citrus aurantium* L.), Houpu (*Magnolia officinalis Rehd. et Wils.*), Muxiang (*Aucklandia lappa Decne.*), Huomaren (*Cannabis sativa* L.), and Dahuang (*Rheum palmatum* L.), is a commonly useful formula for the treatment of PAA in China. Under the guidance of the TCM theory, the IFRD plays an important role in the special therapeutic method of “removing blood stasis and promoting blood circulation.” Additionally, many herbs included in the formulation exert anti-inflammatory effect. Zhou et al. [72] have reported that the IFRD inhibited inflammation, fibrosis, and neovascularization in the progression of PAA. Furthermore, IFRD promotes circulation in the intestine and eventually improves intestinal functional recovery. Wang et al. [73] showed that clinical treatment with IFRD for 3 days after abdominal surgery can promote intestine function recovery. These data indicated that the IFRD may be an effective decoction in the treatment of PAA.

### 3.2. Dachengqi Decoction

Dachengqi decoction (DCQD), a famous preparation of TCM composed of Dahuang (*Rheum palmatum* L.), Houpu (*Magnolia henryi* Dunn), Zhishi (*Citrus aurantium* L.), and Mangxiao (*Natrii Sulphas*), is a commonly used formula for the treatment of postoperative gastrointestinal dysfunction, intestinal obstruction, and pancreatitis [74–76]. A clinical study showed that treatment with DCQT for 1 to 2 weeks could enhance gastrointestinal motility, adjust the synchronized recovery of the alimentary tract, and restore gastrointestinal function after abdominal surgery [75, 77]. DCQD may inhibit inflammatory response and the immune response and improve intestinal injury in the treatment of PAA. Therefore, DCQD is worthy of further research in clinical application.

### 3.3. Sijunzi Decoction

Sijunzi decoction, typically comprised of Renshen (*Panax Ginseng C. A. Mey.*), Baizhu (*Atractylodes Macrocephala Koidz.*), Gancao (*licorice*), and Fuling (*Poria Cocos (Schw.) Wolf.*), has demonstrated effects of invigorating Qi and strengthening the spleen. Sijunzi decoction not only reduces intestinal mucosal injury and promotes the recovery of intestinal function but also improves the adaptive immune function after surgery [78]. A previous clinical study has shown that treatment with Sijunzi decoction for 3 days can further promote the recovery of gastrointestinal function of patients after laparoscopic gallbladder surgery [79]. These observations indicate that Sijunzi decoction is potentially a therapeutic drug for PAA. However, the precise mechanisms of the formula in the treatment of PAA still require further investigation.

### 3.4. Danhong Injection

Danhong injection (DHI) is a Chinese medicinal product approved by the China Food and Drug Administration (CFDA), which is extracted from Danshen (*Radix Salviae*) and Honghua (*Carthami Flos*) [80, 81]. The main bioactive constituents in DHI include salvianic acid A, salvianic acid B, protocatechuic aldehyde, and rosmarinic acid. DHI is a potential antioxidative agent and an anti-inflammatory drug. The anti-inflammatory activity of DHI is primarily due to hydroxysafflor yellow A, and antioxidative capacity relies on salvianolic acid B [82]. Wu et al. [83] have reported that treatment with DHI for 7 days alleviates the formation of abdominal adhesion by inhibiting inflammation, collagen deposition, and oxidative stress in a rat model. DHI has been extensively used in clinical practices for a long time, which supported to serve as a promising drug to prevent PAA [84].

### 3.5. Huoxuetongfu Formula

Huoxuetongfu formula is composed of six crude herbs: Dahuang (*Radix Rhei Et Rhizome*), Taoren (*Persicae Semen*), Yanhusuo (Corydalis Rhizoma), Laifuzi (*Raphani Semen*), Mangxiao (*Sulfas Natrii*), and Honghua (*Carthami Flos*). The Huoxuetongfu formula could play an antiadhesion role through an intestinal mucosal immune barrier, oxidative stress, and inflammatory response [85]. Zhao et al. [86] have reported that the Huoxuetongfu formula alleviates PAA through regulating the suppressor of the cytokine signaling (SOCS)/Janus kinase 2 (JAK2)/STAT/peroxisome proliferator-activated receptor-*γ* (PPAR-*γ*) pathway in the experimental model of Raw264.7. On the basis of experimental research, the Huoxuetongfu formula has been applied clinically in the treatment of PAA [87, 88]. The clinical studies showed that patients with adhesive intestinal obstruction, with the treatment of the Huoxuetongfu formula for 4 weeks, are significantly better than the placebo in gastrointestinal function recovery, which contributes to prevent PAA. The Huoxuetongfu formula is worthy of further research in clinical application.

### 3.6. Daikenchuto

Daikenchuto, a traditional Japanese medicine, consists of Japanese pepper, processed ginger, ginseng radix, and maltose powder. It has been widely used for various intestinal disorders in Japan and the US [89, 90]. In addition to inhibiting inflammatory response, daikenchuto can prevent peritoneal fibrosis by decreasing expression of HSP47, a collagen-specific molecular chaperone, and involved in the progression of peritoneal fibrosis [91, 92]. A previous study indicated that treatment with daikenchuto for 22 days can prevent PAA in the experimental model of mice [93]. Tokita et al. [93] reported that pharmacological modulation of transient receptor potential vanilloid type 1 (TRPV1) might be relevant to the prevention of PAA by daikenchuto. Numerous basic and clinical studies suggest that daikenchuto is beneficial for postoperative complications, including ileus and abdominal bloating [94].

## 4. Acupuncture Treatment

Acupuncture treatment has a wide range of clinical applications in China, which has been considered an effective method for treating gastrointestinal dysfunction diseases for thousands of years [95]. Zusanli (ST36), a confluent acupoint of Yang Ming meridians, is located on the anterior aspect of the lower leg below Dubi (ST35) and one finger-breadth from the anterior crest of the tibia [96]. Studies have shown that electroacupuncture (EA) at ST36 can promote gastrointestinal motility, significantly improve gastrointestinal microcirculation, improve the blood flow and the immune system, and reduce inflammatory injury after surgery [97]. Du et al. [98] also supported that ST36 acupuncture can significantly decrease the expression of inflammatory cytokines, inhibit the activated immune response, and prevent abdominal adhesion formation after surgery via activating the cholinergic anti-inflammatory mechanism. Clinical study showed that the effective rate of acupuncture treatment was 78.7% in 56 patients with postoperative intestinal adhesion [99]. Acupuncture treatment is a unique and effective treatment, which has been generally accepted by the whole world.

## 5. Discussion

PAA, a serious complication accompanied with abdominal surgery, has become a challenging clinical problem owing to the health problems it brings about and a heavy financial burden to the family and society. Therefore, it is important to find some efficient methods to prevent PAA. Chinese medicinal herbs and monomers, herbal formulas, and acupuncture treatment have been demonstrated to have effective effects in the treatment of PAA. Further investigation into their effects, given the prevalence of the complication and demand from patients with PAA for alternative and natural treatment options, is necessary.

Current research demonstrates that compounds isolated from Chinese herbs have beneficial effects for the treatment of PAA, which provides a massive amount of information on natural products. For example, previous research has shown that tanshinone IIA liquid nanoparticles were effective in the treatment of PAA owing to the effects of anti-inflammatory, antioxidative, and modulation of collagen metabolism [100]. In addition, Changtong oral liquid, a Chinese medicine compound in Nanfang Hospital, exerts a protective effect in postoperative intestinal adhesion by modulating the activity of the fibrinolysis system [101]. Ligustrazine nanoparticles nano spray can significantly reduce the incidence of PAA, which may be the activation of the nuclear factor erythroid 2-related factor 2 (Nrf2)—an antioxidant response element (ARE) signaling pathway [6].

## 6. Conclusion

No single approach has been wholly satisfactory in reducing adhesion after surgery. TCM is not only an effective solution for primary health care but also a great resource for drug innovation and discovery, which provide a massive amount of information on natural products [46]. TCM research is providing exciting new points for therapeutic agents. The review brings new insights into further research in the treatment of PAA. Further investigation into herbal formulas and their compounds is therefore warranted.
